# Dyskeratosis congenita associated with a novel missense variant in TERT: Approach for the dermatologists

**DOI:** 10.1007/s00403-024-03050-9

**Published:** 2024-06-28

**Authors:** Constanza Neri Morales, Daniel Cuestas, Felipe Ángel, Felipe A. Ilelaty Urbano, Paula Andrea Rodríguez, José Abraham Brito, Daniel Téllez, Isabel Fernández, Luis Celis Regalado

**Affiliations:** 1 Department of Biosciences, Universidad de La Sabana, Campus Puente Común, Km. 7, Autopista Norte de Bogotá, Chía, Colombia; 2 Estudioderma - Dermatologic Investigation Center - Medical Research Area, Bogotá, Colombia; 3 Department of Medical Genetics Unit, Policlínico Metropolitano, Caracas, Venezuela; 4 Dermatology Program, Universidad El Bosque, Bogotá, Colombia

**Keywords:** Dyskeratosis congenita, TERT, Cutaneous manifestations

## Abstract

Dyskeratosis congenita (DC) is a telomeropathy presenting diagnostic and therapeutic challenges across multiple specialties; yet, subtle dermatological signs enable early detection, altering patient prognosis. A specific DC genetic sequencing was performed according to the clinical criteria of our patient in study. Subsequently, cross-checked information in the main genetic databases was carried out. Additionally, an extensive review of the literature was made to organize the main dermatological aspects in DC. We report a novel variant of DC. Additionally, we share 10 useful and practical messages for dermatologists and any specialist caring for this group of patients.

## Introduction

Dyskeratosis congenita and disorders related to telomere biology (DC/TBD) are caused by poor telomere maintenance resulting in short telomeres [1, 2]. The prevalence of this telomeropathy is 1/1,000,000,000 with a male predominance (13:1) [3, 4]. Given the limited information that exists it can be misdiagnosed. Approximately 9 cases per year have been described from 1910 to 2022 in which the genes ACD, CTC1, DKC1, NHP2, NOP10, PARN, RTEL1, TERC, TERT, TINF2 and WRAP53 are implicated with DC/TBD [5].

This study highlights dermatologic diagnostic clues useful for healthcare professionals to suspect it and how these findings influence genetic testing requests. Additionally, a novel mutation in the TERT gene is reported.

## Methods and case

11-year-old boy with a 9-year history of progressive mucosal and adnexal changes. At 3 years old, he developed tongue architecture alterations, including a tumor-like plaque with a velvety, whitish surface on the left lateral and posterior side, variable nail changes, and light brown reticular hyperkeratosis on the palms. Additional findings included short stature, developmental delay, and periodontal disease.

At 7 years old, he presented with esophageal stenosis, rectal fistula, bone marrow aplasia, and recurrent thrombocytopenia, requiring multiple corticosteroid and cyclosporine treatments. Family history revealed a sister who died of bone marrow aplasia at 17, while parents had no phenotypic abnormalities. Differential diagnoses included 20-nail dystrophy, Nail-patella syndrome, DC, poikiloderma with neutropenia, and other ectodermal dysplasias. Hematological findings raised suspicions of Fanconi anemia, Diamond-Blackfan anemia, and Shwachman-Diamond syndrome.

We considered it unnecessary to perform an exome by mass sequencing, and therefore targeted genetic sequencing was performed in DC/TBD [5]. From genomic DNA extraction, the exome library was prepared by probe capture and PCR enrichment of coding regions and intronic regions (+ 20 bp) adjacent to the exons of approximately 21 thousand genes (Agilent SureSelect Human All Exome V6). The exome library was fed into the Illumina NovaSeq-6000 sequencer and the results were analyzed based on the human genome reference sequence (hg19) and subsequently (hg38) by Varsome [6]. Sequencing, annotation, and variant calling, were performed in Novogene Sample Receiving Admera Health and bioinformatics analysis in collaboration with Sophia Genetics DDM, meeting specific quality criteria. Coverage > 90% and an average depth of 103x were obtained. The targeted panel/exome results only contain findings related to the patient’s clinical history and are analyzed considering OMIM, Ensembl, LOVD, HGMD, ClinVar, gnomAD, Varsome, Beacon Network databases, in silico prediction tools, and American College of Medical Genetics (ACMG) variant classification criteria.

## Results

**Fig. 1:**
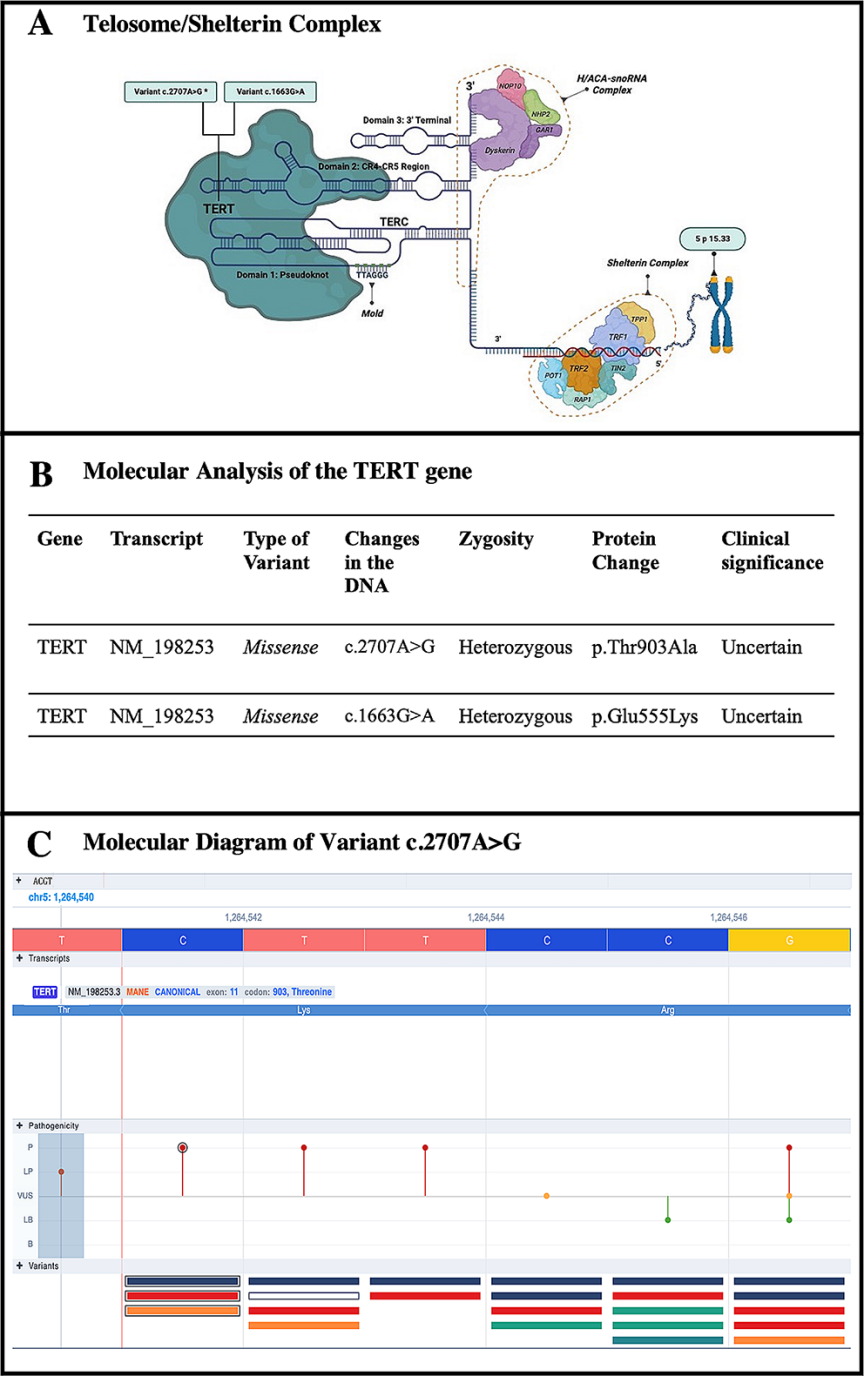
Molecular characteristics of the novel variant. **Panel A** shows the telomerase-shelterin complex which is composed of the H/ACA-snoRN complex (dyskerin, NOP10, NHP2 and GAR1,), TERC consisting of a functional non-coding RNA molecule, composed of 3 domains: 1. Pseudonucleotide, responsible for transporting the nucleotide template. 2. CR4-CR5 region, where it binds to TERT. 3. 3’ terminal, responsible for binding to the proteins of the H/ACA-snoRN complex and finally the Shelterin complex composed of its 6 protein subunits TRF1, TRF2, TPP1, POT1, RAP1 and TIN2, the latter responsible for the stabilization of the telomerase-shleterin complex [21]. **Panel B** shows the molecular analysis of the patient in study. There are two missense variants with uncertain clinical significance c.2707 A > G (TERT: NM_198253, p.Thr903Ala), and c.1663G > A (TERT: NM_198253,p.Glu555Lys) both in the heterozygous state. **Panel C** shows a schematic diagram of the variant c.2707 A > G taken from Varsome® which describes different characteristics of this novel variant mutation: ACGT, Transcripts, Pathogenicity and Variants. Our variant in study is the one located in the thymine (T) base chr5: 1,264,540 in the Threonine amino acid. The variant which is located inside the blue box is classified as LP. Note that the variant has not been described before in databases in comparison with other previous variants [6]. P: Pathogenic LP: Likely Pathogenic VUS: Uncertain Clinical Significance LB: Likely Benign B: Benign.

We identified two variants in the TERT gene (Fig. 1) with uncertain clinical significance (VUS) both in the heterozygous state. The c.2707 A > G variant is a new variant since it has not been reported in the Ensembl, RefSeq, gnomAD, ClinVar, Varsome, or Beacon Network databases. This variant has a strong clinical-genetic correlation with predictors of pathogenicity based on evidence of multiple predictors in silico. The Meta Score by varsome was 10 for moderate pathogenicity (BayesDeladdAF, MetaLR, MetaRNN, MetaSVM, REVEL) and 9 individual predictors for moderate pathogenic variant (CADD, Polyphen2, HDIV, Polyphen2 HAVR, DEOGEN2, EVE, M-CAP) with 3 supporting predictors of pathogenicity (FATHMM, mutation assessor, MVP) (see attached files) [6]. Regarding the c.1663G > A variant, it has already been described in the literature and has not been the subject of our study.

## Discussion

### Clinical workup and update

This genetic disease is defined by the classic triad: dystrophic nails, oral leukoplakia, and reticular pigmentation in the upper thorax and/or neck [7]. Only a portion of DC patients exhibit the full triad, with approximately 80–90% presenting at least one mucocutaneous manifestation [2]. Some patients may also develop systemic complications as subtle phenotypic characteristics emerge gradually [7]. 

### Ungular findings

**Fig. 2:**
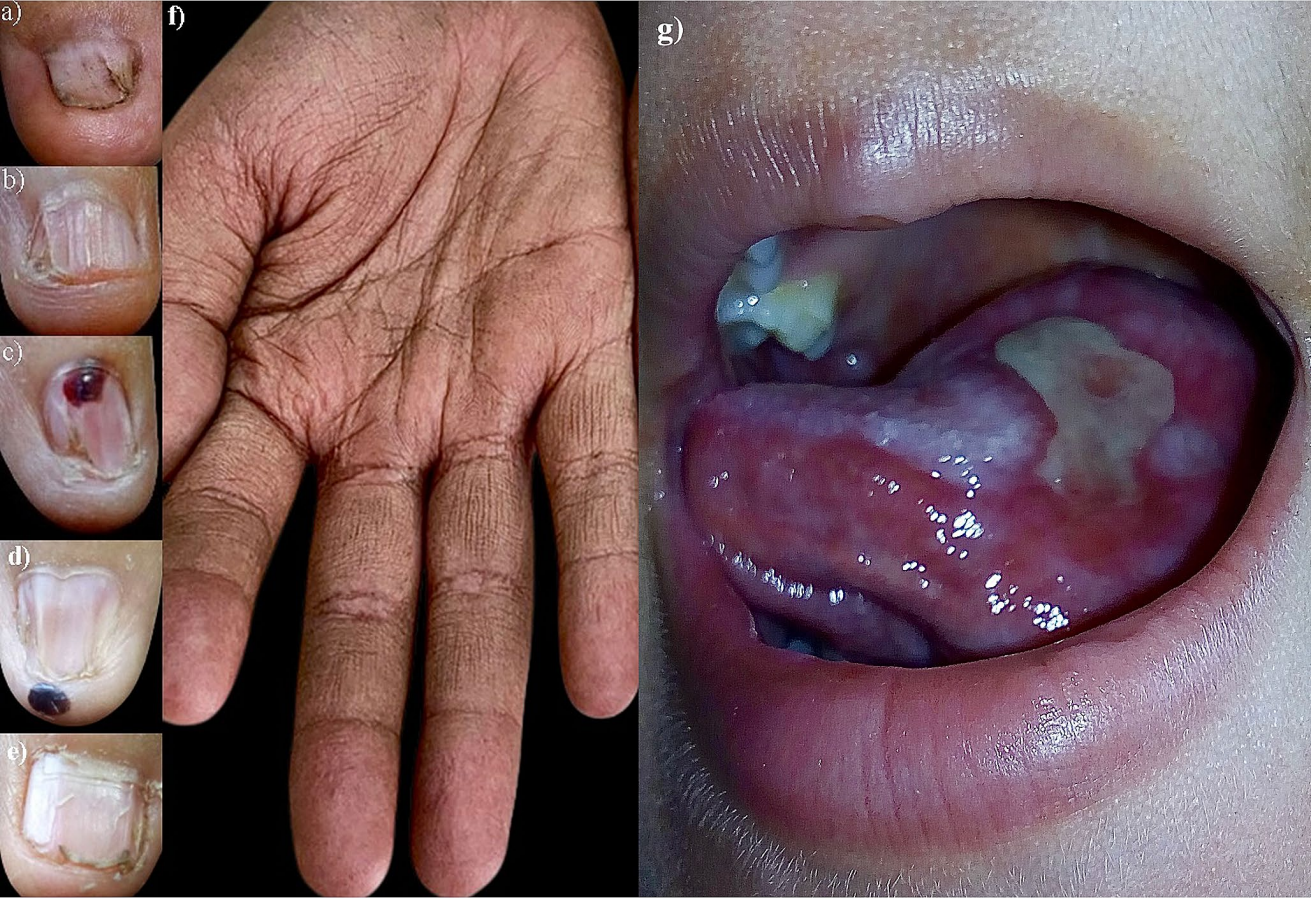
Clinical findings in DC (**a**) Onychodystrophy; (**b**) Trachyonychia; (**c**) Koilonychia; (**d**) Ventral pterygium (**e**) “Boxnail” pattern; (**f**) palmar hyperkeratosis; (**g**) Oral hairy leukoplakia on the tongue. Note subungual and periungual hemorrhages in (**c**) and (**d**) that give clues of hematologic involvement

The involvement can be ungular or periungual (atrophy of the fingertips). Nail findings include anonychia, onychatrophia, onychoschizia, ungueal striae, longitudinal ridges, trachyonychia, koilonychia, ventral and dorsal pterygium, boxnail pattern, clubbing nails, leukonychia and black chromonychia [4, 7]. These findings are reported in up to 88% of cases [3]. Additionally, we found subungual and periungual hemorrhages that give clues of hematologic involvement (Fig. 2).

### Mucosal findings

Mucocutaneous features typically develop between 5 and 15 years of age [3]. The main characteristic is leukoplakia involving the tongue (Fig. 2), oral mucosa, gums, or hard palate (60–80%), with a potential for malignant transformation and it can be detected in the first months of life [3, 8]. Less frequent manifestations are lichen planus, brown pigmentation, papillary and mucosa atrophy, short blunted roots, hypocalcification, taurodontism, periodontitis, early caries, gingival edema and hemorrhage [8]. Histopathology reveals hyperplasia and parakeratotic hyperkeratosis with variable dysplastic changes [4]. The involvement of other mucous membranes includes dysphonia, dysuria, phimosis, epiphora, stenosis of the lacrimal drainage system, blepharitis, ectropion, and entropion [3]. 

### Skin and other adnexal manifestations

In most cases, patients present with grayish-brown hyperpigmentation, or in a minority of cases hypopigmentation, which may be subtle or diffuse, associated with atrophy, and poikiloderma [2–4]. The localization of the lesions usually appears in sun-exposed or flexural areas [4]. 

Other manifestations include palmoplantar hyperkeratosis (Fig. 2), blistering with trauma, chronic ulceration of the fingers and hyperhidrosis prevalent in 15% of cases, which may involve the palmoplantar regions, forehead, or axillae [4, 9]. Hair is also affected leading to fine appearance, alopecia of the scalp, loss of eyebrows or eyelashes, premature graying and trichiasis [4, 10]. 

### Risk of malignant transformation

Telomere shortening increases chromosomal abnormalities, elevating the risk of malignant transformation, with oral mucosa squamous cell carcinoma (SCC) being the most common in DC [10]. SCC develops from oral leukoplakia or erythroleukoplakia of long evolution. Malignant transformation is between 0.13% and 34%, according to the clinical presentation which may be thin, thick/homogeneous, granular, verruciform, or erythroleukoplakic [5, 11, 12]. Less frequently, there is a risk of developing SCC in the nasopharyngeal mucosa, esophagus, vagina, cervix, and rectum [5, 10, 13]. Skin SCCs most commonly occur on the head and neck but can appear anywhere, particularly in sun exposed areas or in patients receiving post-Bone Medular Transplantation (BMT) therapy [10]. 

People with DC tend to develop these malignant tumors at an earlier age compared to the general population [1, 10]. The average age of diagnosis of SCC is between 20 and 30 years, where most of the reports are concentrated before a BMT, for basal cell carcinoma, its report is restricted to events after BMT, being less frequent [5].

### Graft-versus-host disease

BMT recipients are at risk of developing cutaneous graft versus host disease (GVHD), mimicking poikiloderma and nail changes seen in dyskeratosis. Chronic GVHD initially presents as violaceous erythema, progressing to lichen planus or sclerodermiform lesions. This inflammatory condition, often alongside hyperpigmentation, blistering, nail dystrophy, and alopecia, complicates the differential diagnosis with DC [10, 14]. The suspicion of acute or chronic GVHD should be presumed according to generalized skin involvement after BMT and histopathological support [10, 14, 15]. Our patient has not yet received BMT.

### Biomolecular background

The diagnostic suspicion of DC is clinical, and its confirmation is molecular [7]. Vulliamy et al. proposed diagnostic criteria for DC, categorized into major (classic triad) and minor criteria, detailed in Table 1^7^. Our patient meets two criteria of the classic triad and five of the minor criteria.

The presentations of this telomeropathy arise through recessive inheritance, heterogeneous dominant, X-linked, or by de novo mutation generating direct effects on telomerase [16]. In our case, we found an autosomal recessive inheritance. The proportion of telomeropathies associated with DC due to TERT is described between 1 and 7% for autosomal dominant and/or recessive forms [17–20]. Telomerase is a holoenzyme that is responsible for maintaining telomere length of stem and germ cell chromosomes, preventing cellular senescence and apoptosis [21].

Telomerase consists of a ribonucleic component (TERC) with 451 ribonucleotides, acting as a template for adding TTAGGG to telomeres’ 3’ ends for elongation, and an enzymatic component, telomerase reverse transcriptase (TERT) (Fig. 1) [21]. This complex promotes the synthesis and stabilization of TERC, favoring telomeric genesis [21, 22]. 

Mutations in the TERC gene are believed to be an important cause of the occurrence of the autosomal dominant form of dyskeratosis congenita [7, 15, 16, 22–24]. DKC1 (dyskerin) is the most frequently reported mutation (20-25%) in DC, in contrast to the 1–7% described in the TERT gene, highlighting this rare case [7].

The variants identified in this case, c.2707 A > G and c.1663G > A, both in heterozygous state in the TERT gene (5p15.33) are associated with autosomal dominant (AD) and autosomal recessive (AR) dyskeratosis congenita, pulmonary fibrosis and/or bone marrow failure, telomere-related (AD) and acute myeloid leukemia (somatic and AD) (OMIM: 187,270). The c.2707 A > G variant is not reported in the public databases Ensembl, RefSeq, gnomAD, ClinVar, or Varsome nor the literature, its minor allele frequency (MAF) is estimated to be less than 1%, suggesting that the described change may have a negative effect on the phenotype due to its low frequency in the general population [6]. This is a missense variant whose DNA change results in the substitution of the amino acid threonine for alanine, which are residues with different physicochemical properties [6]. The p.Thr903Ala change occurs at a moderately conserved position among species (PhyloP) and 17 of 19 bioinformatic predictors classify it as pathogenic (Varsome) [6]. Therefore, the structure/function of the protein could be affected. Varsome PP2 helps support this variant as probably pathogenic [6]. These scores and rules follow the ACMG and the bioinformatic predictors estimated that it could have a pathogenic effect with a high probability and point to a de Novo mutation (see attached files) [6, 25]. 

Regarding both variants being missense mutations, based on the ACMG guidelines, since benign missense mutations are rare, a new missense mutation can be seen as evidence supporting its potential to cause disease, with a likelihood of being moderately or highly pathogenic [25].

Both identified variants are VUS and require classification. Determining their phase (compound heterozygosity or simple heterozygosity) is crucial, suggesting future parental molecular analysis to elucidate their role in DC development. Nevertheless, genetic predictor results and clinical features strongly indicate pathogenicity in this case.

### Limitations and precisions

Our methodology’s limitations include focusing on point mutations in exonic or splicing regions, and small deletions or insertions. Large chromosomal rearrangements, deep intronic mutations, and variants in regulatory regions may be missed. Additionally, variants in unanalyzed or repeat expansion regions, as well as low-frequency mosaicisms, may not be reliably detected. Only pathogenic, possibly pathogenic, or VUS variants are reported; benign variants are excluded. Variant classification may change with scientific advances. A negative result doesn’t rule out untested mutations.

## Conclusions and highlights

This novel TERT gene variant and its dermatologic manifestations represent the first unreported case globally, making it a valuable addition to databases. We provide 10 practical messages for dermatologists and specialists caring for these patients.

(I) All patients aged 5–15 with confirmed bone marrow aplasia should undergo dermatological assessment for potential genodermatosis manifestations. (II) Given the risk of periodontal disease, caries, and oral malignant neoplasms, close follow-up every 6 months by dentistry is recommended. (III) If the patient undergoes BMT, sources of latent oral infection of dental and periodontal origin should be eliminated. (IV) An annual evaluation to rule out neoplasms, stenosis, hepatic, pulmonary fibrosis, and corneal abrasions is recommended. (V) Simple blood tests are suggested every 6 months to look for bone marrow aplasia. (VI) Early hematologic involvement should be suspected in subjects with subungual and periungual hemorrhages. (VII) In all patients older than 20 years, SCC should be ruled out. (VIII) Dermatologists should be trained in the recognition of acute and chronic manifestations associated with GVHD. (IX) Patients must avoid carcinogenic activities like smoking, alcohol consumption, and unprotected sun exposure; prioritizing photoprotection with physical and chemical measures, even indoors. (X) Not all patients comply with the typical localization of the classic triad so a thorough physical examination by dermatology should be performed [1–25]. 

**Table 1:** Diagnosis Criteria for Congenital Dyskeratosis [5, 7, 20]

Major Criteria
Hyper/hypopigmentation reticular, subtle, and diffuse on the upper chest and/or neck. Nail dysplasia: grooves, scaling, poor growth, partial nail loss. Oral leukoplakia
**Minor Criteria**
Progressive bone marrow failure Solid tumors (in individuals under 50 without risk factors) Myelodysplastic syndrome or acute myeloid leukemia Shortened telomeres: assessed by telomere length through Flow-FISH Epiphora, blepharitis Excessive dental caries, tooth loss, taurodontism Premature hair loss, appearance of gray hair, sparse or altered eyelashes Hyperhidrosis Intrauterine growth retardation Short stature for age Mental retardation Developmental delay Peptic ulcer, gastrointestinal telangiectasias, esophageal stenosis, hepatopathy Ataxia, cerebral hypoplasia, microcephaly Hypogonadism, undescended testicles, phimosis, urethral stenosis Osteoporosis, avascular necrosis, scoliosis Deafness Avascular necrosis of the hip or shoulder
**Diagnostic Possibilities**
Two major criteria. One major criteria + 2 or more minor criteria. One feature of the triad or suggestive family history: appearance of bone marrow failure (BMF), myelodysplastic syndrome (MDS), acute myeloid leukemia (AML), early-onset squamous cell carcinoma of the head/neck (HNSCC), and/or pulmonary fibrosis (PF) in a first- or second-degree blood relative in combination with: a) Progressive BMF. Can occur at any age and may be a presenting sign. Macrocytosis and elevated levels of hemoglobin F may be observed. b) MDS or AML. Can be a presenting sign. c) Solid tumors, usually HNSCC or anogenital adenocarcinoma, in individuals under 50 years of age without other risk factors. Solid tumors may be the first manifestation in individuals without BMF. d) Pulmonary fibrosis.”

## Data Availability

Data is provided within the related files.
